# Supplementary material for: The impact of alloying on defect-free nanoparticles exhibiting softer but tougher behavior
Source: Nat Commun. 2021 May 4;12:2515. doi: 10.1038/s41467-021-22707-x (PMC8096810; doi:10.1038/s41467-021-22707-x)
Supplement: Supplementary file 1 — Supplementary Information [file 41467_2021_22707_MOESM1_ESM.pdf]

Supplementary Information

The impact of alloying on defect-free nanoparticles exhibiting softer but tougher behavior

A. Bisht et al.

Supplementary Figures

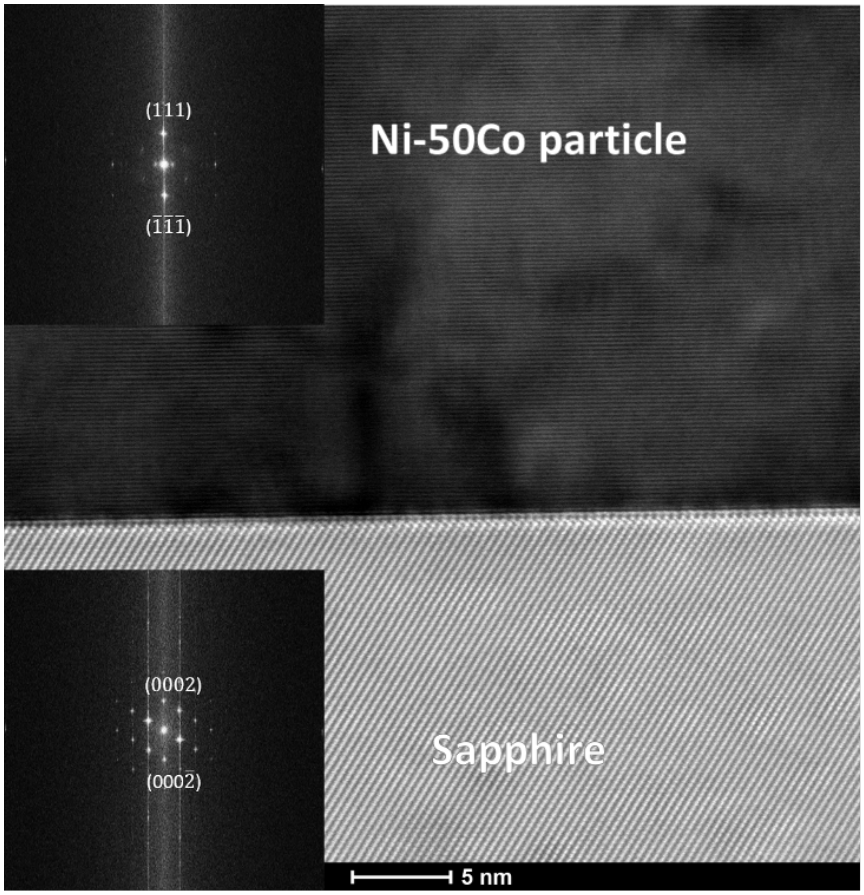

Supplementary Figure 1 Characterization of the particle-substrate interface. HR-STEM micrograph showing the interface between the Ni-0.5Co particle and the substrate. The fast-Fourier transform (FFT) confirms that the (111) particle plane is parallel to the (0002) substrate plane and that no intermetallic phases are present at the interface.

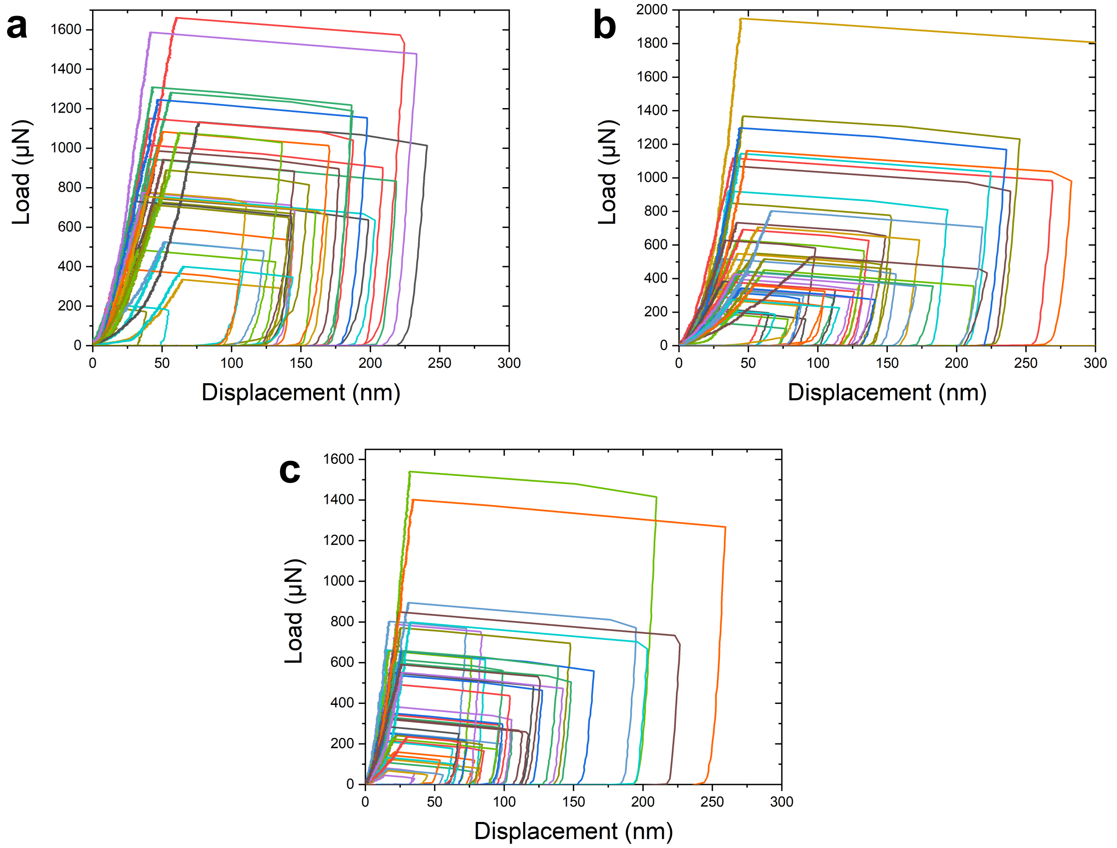

Supplementary Figure 2 Mechanical testing of nanoparticles. The complete set of load-displacement curves for all particles exhibiting strain burst during compression tests. **a** Ni, **b** Ni-0.3Co, **c** Ni-0.5Co

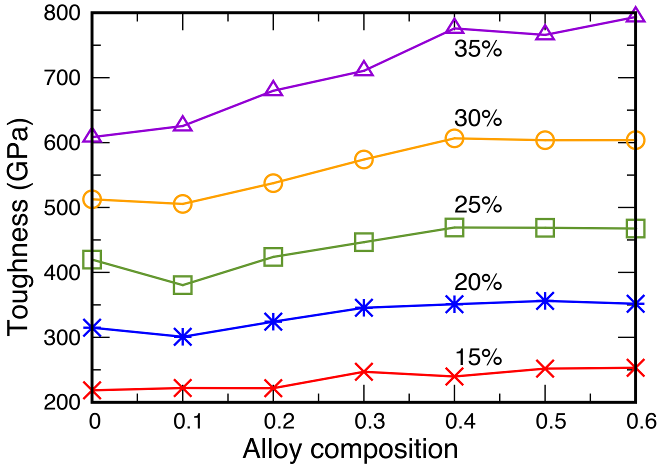

Supplementary Figure 3 Alloying increases the toughness of nanoparticles. The toughness of 35 nm nanoparticles as a function of chemical composition computed for several stains indicated by the labels.

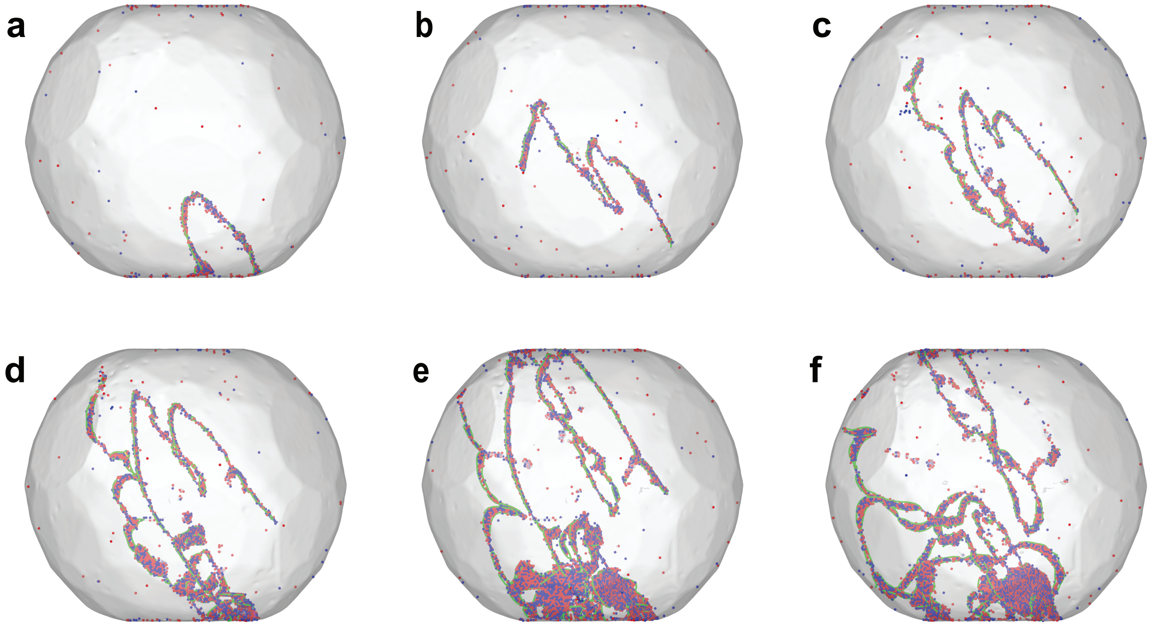

Supplementary Figure 4 Evolution of dislocation configurations in a 35 nm Ni-0.5Co nanoparticle. **a-d** Sequential snapshots of the particle. **a-c** A single dislocation nucleates at the lower facet and propagates deeper into the particle. **d** The dislocation reaches the top and bottom facets, and **e**,**f** causes the nucleation of new dislocations.

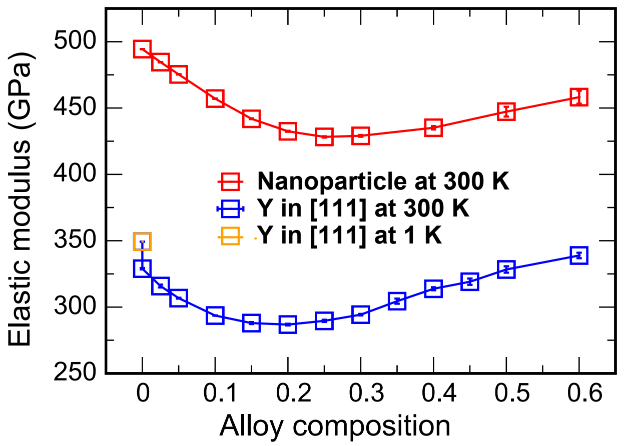

Supplementary Figure 5 Elastic properties of alloy nanoparticles. Effective elastic modulus of 35 nm nanoparticles as a function of chemical composition compared with the ideal Young modulus $Y_{[111]}$ in the [111] direction at 300 K. The pure Ni Young modulus $Y_{[111]}$ at 1 K is shown as a reference.

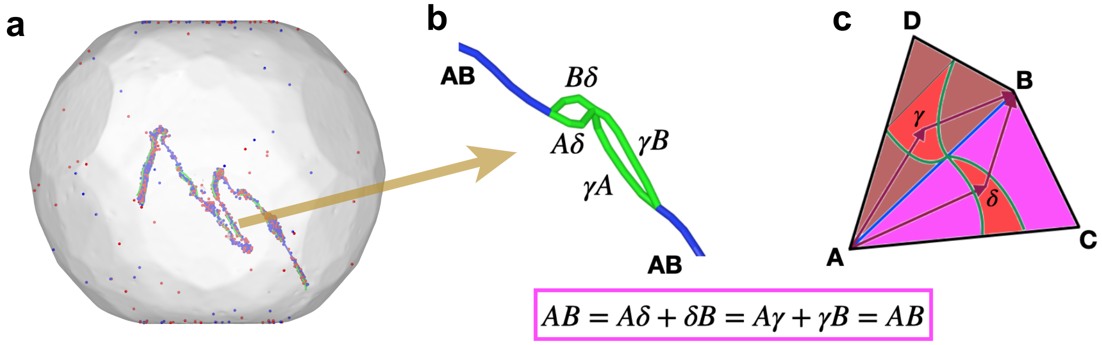

Supplementary Figure 6 Dislocation cross-slip in Ni-Co nanoparticles. **a** Zigzag-shaped dislocation in a 35 nm Ni-0.5Co nanoparticle. **b** Dislocation segment of a full dislocation (blue) dissociates into partials (green) in two different slip planes. The visualization is implemented by the DXA algorithm using OVITO [?]. **c** Thompson tetrahedron explaining the crystallography of the cross-slip. The dislocation reaction is shown in the frame.

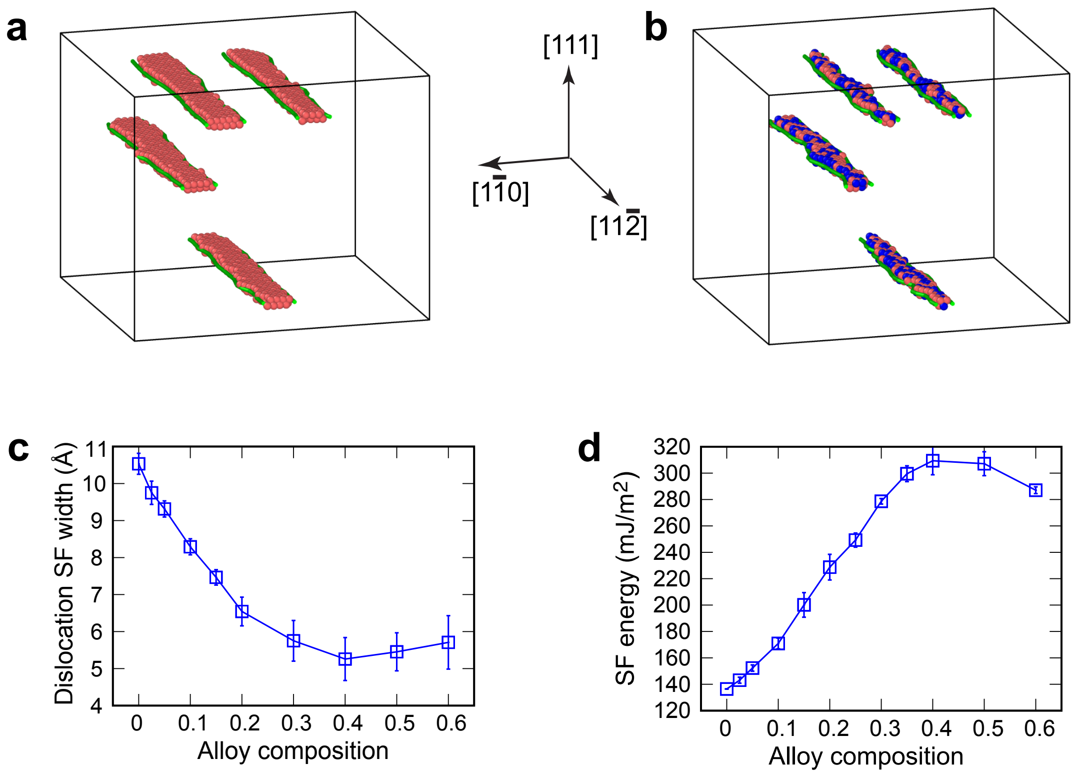

Supplementary Figure 7 Alloying effect on the stacking fault energy. Edge dislocations in **a** pure Ni and **b** Ni-0.5Co random alloy. **c** Stacking fault width in the dissociated dislocations as a function of Co concentration. **d** Stacking fault energy as a function of alloy concentration.
